# How Do Mesenchymal Stem Cells Influence or Are Influenced by Microenvironment through Extracellular Vesicles Communication?

**DOI:** 10.3389/fcell.2017.00006

**Published:** 2017-02-07

**Authors:** Gabriel Dostert, Benjamin Mesure, Patrick Menu, Émilie Velot

**Affiliations:** ^1^Laboratoire d'Ingénierie Moléculaire et Physiopathologie Articulaire, UMR 7365 Centre National de la Recherche Scientifique – Université de Lorraine, Biopôle de l'Université de LorraineVandœuvre-lès-Nancy, France; ^2^Faculté de Pharmacie, Université de LorraineVandœuvre-lès-Nancy, France

**Keywords:** mesenchymal stem cells (MSCs), extracellular vesicles (EVs), recipient/donor cells, intercellular communication, microenvironment

## Abstract

Mesenchymal stem cells (MSCs) are widely used in cell therapy and tissue engineering thanks to their self-renewal, their multipotency, and their immunomodulatory properties that make them an attractive tool for regenerative medicine. A large part of MSCs positive effects is due to their secretion products which participate in creating a favorable microenvironment and closely relate these cells to other cell types. Extracellular vesicles (EVs) belong to cellular secretions. They are produced by cells continuously or after stimulation (e.g., calcium flux, cellular stress) and act in tissue homeostasis and intercellular communication. The understanding of the role of EVs is growing, more particularly their impact on cell migration, differentiation, or immunomodulation. EVs derived from MSCs show these interesting properties that may be considered in therapeutics, although they can have adverse effects by facilitating cancer propagation. Moreover, MSC behavior may also be influenced (proliferation, differentiation) by EVs derived from other donor cells. The aim of this mini review is to summarize the two-way communication between MSCs and other cell types, and how they can affect each other with their microenvironment through EVs. On the one hand, the manuscript presents the influence of MSC-derived EVs on diverse recipient cells and on the other hand, the effects of EVs derived from various donor cells on MSCs. The discrepancies between cancer cells and MSCs communication according to the sources of MSCs but also the tumor origins are also mentioned.

## Introduction

At physiological level, the cells composing a tissue, an organ, or even an entire organism are constantly trading information either by physical contact or by long distance communication. This phenomenon allows their maintenance but can also lead to variations in the cellular behavior. The study of these interactions has permitted to develop new therapeutic strategies such as cell therapies.

Thanks to their self-renewal, their multipotency, and their immunomodulatory properties, mesenchymal stem cells (MSCs) are an attractive tool for regenerative medicine. For example, MSCs are used in clinical trials to treat pathologies such as graft vs. host disease (GvHD) (Le Blanc et al., 2008; Zhou et al., 2010; Introna et al., 2014), although concerns remain regarding efficacy, safety, and feasibility of such treatments (Si et al., 2011; Mendicino et al., 2014). The most common sources of MSCs are from adult origin like bone marrow or adipose tissue, but their collects request an invasive procedure. Perinatal sources like Wharton's jelly from umbilical cord offer more accessibility and larger quantities of MSCs with higher proliferation rate and greater immunomodulatory properties (El Omar et al., 2014).

Numerous studies have shown the regenerative potential of MSCs to counteract organ failures (Xing et al., 2014; Desando et al., 2016; Sattayaprasert et al., 2016). It has also been demonstrated that injecting MSC conditioned medium and not the cells themselves can induce the same effects. This is due to the composition of MSC secretions, which are of two types, soluble factors [e.g., soluble tumor necrosis factor receptor 1, transforming growth factor (TGF-β) 1; (Melief et al., 2013; Ke et al., 2016)] or extracellular vesicles (EVs). EVs are involved in tissue reparation, immunomodulation, and proliferation. A miscellaneous EV population can be found in biological fluids. Three kinds of EVs are mostly described by the scientific community according to their size and biogenesis (Höög and Lötvall, 2015; Yáñez-Mó et al., 2015; Kowal et al., 2016). The biggest vesicles are secreted after cell apoptosis and are large EVs from 1 to 5 μm called apoptotic bodies (Atkin-Smith et al., 2015). EVs from 0.1 to 1 μm are termed microparticles, ectosomes, or microvesicles. They are generally produced by cells during stress or metabolic changes and result from the budding of the plasma membrane (Ratajczak et al., 2006). Endosome-derived EVs named exosomes are small EVs—with a size varying from 30 to 150 nm depending on the literature—which are secreted continuously whatever cellular state (stress or physiological conditions) (Valadi et al., 2007; György et al., 2011; Vlassov et al., 2012; Crescitelli et al., 2013). The size similarity between EVs can impair their identification if size is used as the only parameter for characterization. Lately, the difficulties to purify a homogeneous EV population and to select appropriate EV markers to classify them have been discussed and remain. All vesicle populations will be referred as EVs in this mini review and the attention will be highlighted on their biological effects.

**Figure 1:**
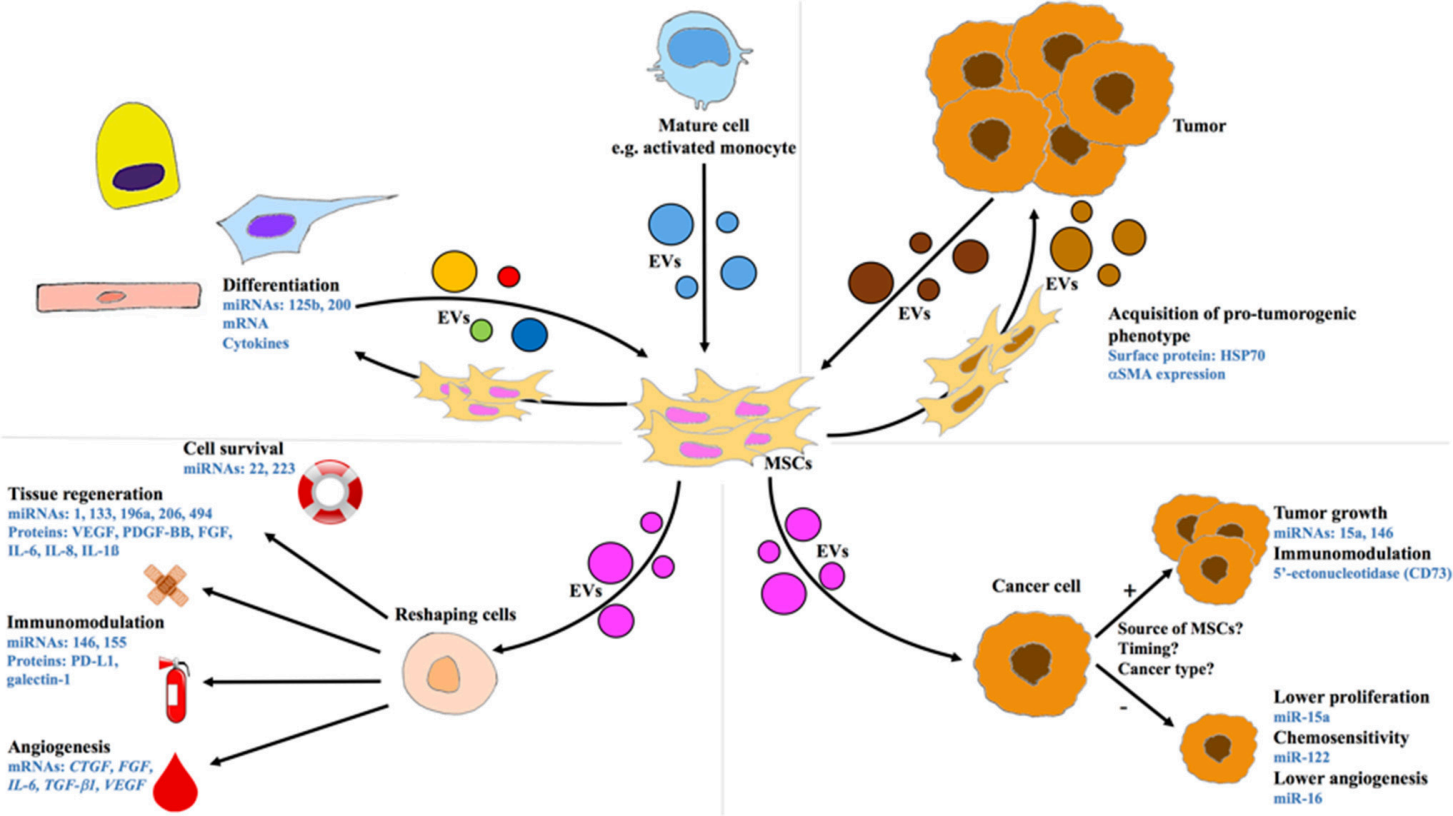
**Intercellular communication between MSCs and other cell types through EVs**. **Upper panels:** MSCs as recipient cells. **Lower panels:** MSCs as donor cells. **Left Panels:** MSCs exchanging with differentiated cells. **Right Panels:** Cross-talk between MSCs and cancer cells. CTGF, connective tissue growth factor; EV, extracellular vesicle; FGF, fibroblast growth factor; HSP, heat shock protein; IL, interleukin; mRNA, messenger RNA; miRNA or miR, microRNA; MSC, mesenchymal stem cell; PDGF, platelet-derived growth factor; PD-L1, programmed death-ligand 1; SMA, smooth muscle actin; TGF, transforming growth factor; VEGF, vascular endothelial growth factor.

MSCs are commonly known as donor cells by providing EVs to other cells types, nevertheless their part as recipient cells is less described (Figure 1). A recent report shows that human bone marrow (BM)-MSCs can be both donor and recipient cells. Osteogenically induced BM-MSCs are donors of EVs which are able to guide osteogenic differentiation. The stimulation of recipient BM-MSCs by these EVs induces expression of bone morphogenic protein (*BMP*)*-2, osterix*, osteopontin (*OPN*), osteocalcin (*OCN*), and alkaline phosphatase (*ALP*). Thus, the recipient BM-MSCs may be employed for therapeutic use to improve bone regeneration (Martins et al., 2016). Each cell produces its own secretions which allow the formation of its own microenvironment affecting surrounding cells including MSCs. In this mini review, we focus on the effects of EV exchange between MSCs and other cell types in both ways.

## MSCs as donor cells

### MSC-EVs and tissue regeneration

MSC microenvironment acts differently depending on the target cells (Figure 1). MSC-EVs can produce modifications in gene expression and mediate cell maturation or differentiation. For example, osteoblasts undergoing stimulation with EVs originated from BM-MSCs show clear calcium deposits *in vitro* in the same way as osteogenic medium stimulation. This is supported by *ALP, OCN, OPN*, and runt-related transcription factor (*RUNX*)*2* gene overexpression. These *in vitro* results were confirmed *in vivo* by using hydrogels supplemented with EVs to improve bone regeneration through the expression of micro RNA miR-196a.

MSC-EVs induce angiogenesis stimulation of human umbilical vein endothelial cells by enhancing tube formation in Matrigel™-coated wells. This observation has been made with fresh or frozen EVs, showing their preservation potential under −80°C (Teng et al., 2015). Montemurro et al. showed that EVs derived from cord blood MSCs carry transcripts related to angiogenic and proliferative function such as connective tissue growth factor (*CTGF*), fibroblast growth factor (*FGF*), interleukin (*IL*)*-6* but also *TGF*-ß*1*, and vascular endothelial growth factor (*VEGF*) (Montemurro et al., 2016). Transcripts were not the only ones to participate in the pro-angiogenic properties of MSC-EVs. Pro-angiogenic properties beneficial for myocardial tissue repair were highlighted after MSC pre-conditioning by serum starvation plus low oxygen settings in order to mimic ischemic conditions after myocardial infarction. Modification of culture conditions leads to modulation of protein targeting toward MSC-EVs. Specific protein content during stimulation has an effect in angiogenesis and especially by nuclear factor (NF)-κB signaling pathway (Anderson et al., 2016). Intramyocardial injection of MSCs or MSC-EVs also reduces myocardial ischemia in rats 48 h after infarction. Compared to the control, MSCs or MSC-EVs showed similar effects with reduction of the infarct size 28 days after post-operation. Injection of EVs derived from MSCs also improved cardiac function and promoted the number of blood vessels in myocardium infarcted region with a higher increase than MSC injection (Bian et al., 2014). In skeletal muscle, MSC-EVs are able to improve tissue repair. The specific content in growth factors (VEGF, fibroblast growth factor, platelet-derived growth factor-BB), cytokines (IL-1β, IL-6, IL-8), and especially in non-coding RNA may act to maintain a healthy and functional tissue. Three important myogenic micro RNAs (miRNAs: miR-1, -133, and -206) that have been involved in muscle regeneration after injection in rat model appear inside MSC-EVs. Although miR-494 is not specific for muscle repair, it was also found in high concentration inside MSC-EVs. This miRNA participates in myogenesis, migration activity, and protects against ischemia/reperfusion injury in cardiac muscle (Nakamura et al., 2015; Hofer and Tuan, 2016). The potential of MSC-EVs to treat cardiovascular injuries is widely studied. During ischemia, tissue oxygenation becomes almost null, but during this time, MSCs secrete large amounts of EVs containing a high concentration of miR-22 compared to normoxic conditions. Interactions between EVs containing miR-22 and cardiomyocytes allows a reduction of infarcted zone volume and apoptosis in the ischemic myocardium by the down regulation of methyl CpG binding protein 2 (Mecp2) (Feng et al., 2014). Another study highlights the beneficial effect of MSC-EVs on cardiomyocytes survival, this effect being higher when MSCs overexpress transcription factor GATA-4 (Yu et al., 2015). Wang et al. have pointed miR-223 in MSC-EVs as a primordial effector in cardioprotective properties. During polymicrobial sepsis, miR-223 contained inside EVs contributes to the regulation of two proteins, semaphorin-3A (Sema3A) and signal transducer and activator of transcription 3 (Stat3), which are involved in apoptosis. The absence of miR-223 inside MSC-EVs has an impact on cardiac cells and may have deleterious effects. On the contrary, his presence will allow a better defense against sepsis (Wang et al., 2015). Furthermore, MSC-EV regenerative potential has been demonstrated in neurogenesis (Xin et al., 2013), liver fibrosis (Li et al., 2013), and cutaneous wound healing (Zhang et al., 2015).

### MSC-EVs and immunomodulation

MSC-EVs take place in immunomodulation to lower the immune system activation through the induction of anti-inflammatory cytokines and regulatory T cells (Treg) (Figure 1) (Del Fattore et al., 2015), but also by regulating macrophages polarization (Ti et al., 2015) and neutrophils mobilization (Zhu et al., 2014). More generally, MSC-EVs have been shown to balance expansion of myeloid progenitors (Goloviznina et al., 2016). MSC-EVs can activate monocytes by Toll-like receptor (TLR) signaling pathway. In contrast with activation by lipopolysaccharides (LPS), the surface receptor involved is unknown but will cause the same signaling cascade via myeloid differentiation primary response gene 88 (MYD88) and NF-κB (Zhang et al., 2014). Another notable difference is observed in cytokine production. Thus, when monocytes are stimulated by MSC-EVs, they differentiate into macrophages which secrete IL-10, leading to Treg expansion. During this study, test of skin allograft rejection by mice treated with MSC-EVs showed similar results with another experiment using cyclosporine A, an immunosuppressor (Zhang et al., 2014). MSC-EVs can induce the decrease of B lymphocyte and natural killer (NK) cell proliferation. Unlike MSCs, EV immunomodulation is not mediated by indoleamine-pyrrole 2,3-dioxygenase (IDO) pathway but by a molecule on their surface: programmed death-ligand (PD-L)1 (Di Trapani et al., 2016). To be activated, MSCs need to be stimulated by pro-inflammatory cytokines to trigger their immunosuppressive answer. Generally, an increase of IDO activity is a marker of activation when MSCs and T cells are in contact, but during contact between MSC-EVs and T cells, IDO concentration remains stable without modifying the immunological potential. In addition to PD-L1, an endogenous leptin found on EV surface—galectin-1—is also involved in the immunomodulatory response (Del Fattore et al., 2015). Effects of 5′-ectonucleotidase (CD73) has been studied as well because this enzyme found on MSCs and MSC-EVs actively produces adenosine, a molecule known to be immunosuppressive (Kerkelä et al., 2016). Moreover, inflammatory priming induces the increase of miR-155 and -146 level inside MSC-EVs. These specific miRNAs intervene in the regulation of inflammatory reactions (Di Trapani et al., 2016). Clinical applications are also possible to reduce inflammation in some pathologies such as therapy refractory GvHD. Shortly after MSC-EV therapy, cutaneous, and mucosal GvHD showed a very promising response, allowing to reduce four times the administrated steroid doses. Such EV-based treatment have beneficial effects for the patient without side effects (Kordelas et al., 2014).

### MSC-EVs in cross-talk with cancer cells

Despite the numerous studies about pro-angiogenic effects of MSC-EVs, these vesicles may have a reverse effect on cancer cells. A study on mouse breast cancer cell line (4T1) headed by Lee et al. showed that miR-16 level in EVs derived from mouse BM-MSCs contributes to decrease the secretion of VEGF by cells. These modifications lead to a suppression of angiogenesis *in vitro* and thus a reduction in tumor spread (Lee et al., 2013). In the case of cancers therapy with MSC-EVs, there are a lot of divergences between studies because of the source of MSCs, the tumor origin, but also the timing of stimulation with EVs (Figure 1). Depending on the papers, MSC-EVs can promote tumor progression (Roccaro et al., 2013; Shi et al., 2016), decrease it (Bruno et al., 2013), or have no effect (Hendijani et al., 2015). In all cases, the content in cytokines and miRNAs seems to be the key factor. The effect of nucleic acids contained in EVs in the cross-talk between tumor cells and MSCs has been well-described by Lopatina et al. (2016). They came to the conclusion that these cells exchange either oncogenic and anti-tumoral RNAs. For example, EVs secreted by multiple myeloma BM-MSCs display a lower tumor suppressive miR-15a content (Roccaro et al., 2013), associated with more cytokines regulating cell adhesion and migration as well as oncogenic proteins. When stimulated with MSC-EVs, cells from nasopharyngeal carcinoma (CNE2) undergo toward a mesenchymal transition, with a decrease of epithelial markers like epithelial cadherin (Shi et al., 2016). MSC-EVs used on different cancer cell lines can also stimulate metalloproteinase (MMP)-2 expression, thus helping tumor migration (Yang et al., 2015). These vesicles have also the ability to transfer CD73 on tumor cells, giving them the ability to metabolize AMP into adenosine, which reduces NK and T cell activation. MSC-EVs can also confer drug resistance to gastric cancer cells by stimulating multidrug resistance protein expression and reducing chemo-induced apoptosis (Ji et al., 2015). However, liver cancer cell lines like HepG2 stimulated with MSC-EVs have difficulties in the cell cycle progression and are subject to apoptosis (Bruno et al., 2013). *In vivo* growth of glioma xenografts is also reduced by miR-146 which is present in MSC-EVs (Katakowski et al., 2013). The chemosensitivity of hepatocellular carcinoma cells was also raised by miR-122 contained in adipose-tissue MSC-EVs. Some cell lines remain unaffected by MSC-EVs, neither in a pro- nor an anti-tumor way. For example, when the lung cancer cell line A549 is exposed to umbilical cord MSCs conditioned medium, it does not lose or gain proliferation rate, even if MSCs are stimulated with interferon γ. The association of conditioned medium with the therapeutic agent doxorubicin does not modify its native effect (Lou et al., 2015). Even if the direct use of MSC-EVs in cancer therapies is still not fully understood and should be carefully controlled, it appears that they could be an interesting vector to address therapeutic cargos to tumors (Chen et al., 2011; Johnsen et al., 2014; Yang et al., 2015).

## MSCs as recipient cells

### EVs from differentiated cells

EVs derived from differentiated cells are able to modulate MSC phenotype (Figure 1). An *in vitro* study has highlighted the potential of EVs derived from neuronal cells to mediate MSC neuronal induction. Indeed, miR-125b—which is known to act in neuronal differentiation—is expressed by MSCs after 1 week of stimulation by these EVs (Takeda and Xu, 2015). When derived from endothelial cells, EVs can influence MSC proliferation, migration, and secretion of soluble factors such as matrix MMP-1, MMP-3, chemokine ligand 2 (CCL-2), and IL-6 (Lozito and Tuan, 2014). In the case of renal tubular morphogenesis and kidney structure, cells known as mesenchymal-epithelial cells are required. The origin of these cells can be explained by the migration and transition of MSCs from bone marrow induced by EVs derived from human renal proximal tubular epithelial cells. The presence of the miR-200 family (miR-200a, -200b, and -200c) has been highlighted in these EVs. Their uptake by BM-MSCs induces a MSC phenotype modification with a mesenchymal to epithelial transition, characterized by the acquisition of polarized epithelial cell properties by BM-MSCs. This is a physiological process involved in kidney formation (Chiabotto et al., 2016). Immune cells such as monocytes communicate also with MSCs via EVs. LPS activated monocytes secrete a lot of soluble factors as well as EVs. The conditioned medium of these cells has the property to modulate MSC phenotype by upregulating osteogenic gene expression (Omar et al., 2011). *RUNX2, BMP-2*, and *OCN* expression were evaluated after 72 h of MSC stimulation by conditioned medium or EVs derived from activated monocytes compared to control. During EVs stimulation, *RUNX2* and *BMP-2* were significantly increased compared to control in the same way as conditioned medium but *OCN* was only over expressed with EVs. This indicates that EVs derived from activated monocyte promote osteogenic differentiation in MSCs (Ekström et al., 2013). In a rat model of calvarial bone defect, it has been shown that EVs obtained from induced pluripotent stem cell-derived MSCs associated to a tricalcium phosphate scaffold are able to stimulate bone regeneration by recruiting BM-MSCs at the defect site. The rat's BM-MSCs are activated through the phosphatidylinositol 3 kinase (PI3K)/Akt pathway leading to osteogenic differentiation (Zhang et al., 2016).

### EVs from cancer cells

Genetic modifications of MSCs are also possible via EV transfer from cancer cells (Figure 1). When EVs are originated from healthy cells, their effects seem beneficial. However, when EVs come from cancer cells, their influence on MSCs may be harmful. Lindoso et al. have demonstrated that EVs derived from renal cancer stem cells can induce epigenetic changes in recipient cells. MSCs are attracted to the tumor region and change their phenotype, becoming pro-tumorigenic. This correlates with the overexpression of genes involved in cell migration: *chemokine receptor type (CXCR)4* and *CXCR7*; matrix remodeling: *collagen type IV alpha 3 chain (COL4A3)*; as well as angiogenesis and tumor growth: *IL-8, OPN*, and *myeloperoxidase*. EVs secreted by cancer stem cells allow a better chemoattraction of MSCs, which promote tumor development and spread (Lindoso et al., 2015). There are also differences in the evolution of MSC phenotype which is reliant on the origin of EVs. Thus, MSCs can differentiate into myofibroblasts under action of EVs from prostate cancer. The myofibroblastic marker alpha-smooth muscle actin α-SMA) is expressed by more than 50% of MSCs exposed to prostate cancer derived EVs vs. only 5% of cells under TGF-ß1 stimulation, which is known to induce α-SMA expression. There is also a correlation between the quantity of EVs and α-SMA acquisition (Chowdhury et al., 2015). This differentiation has also been demonstrated with EVs from breast cancer cells or chronic lymphocytic leukemia (Cho et al., 2011; Paggetti et al., 2015). Another example of MSC phenotype modification has been highlighted by Li et al. during a study about lung tumor EVs. *In vitro*, EVs from the lung cancer line A549 stimulate the production and secretion of inflammatory cytokines in MSCs. Three cytokines: IL-6, IL-8, and monocyte chemoattractant protein (MCP)-1 were released by MSCs when they are triggered by A549 cell-derived EVs. The priming of MSCs by lung cancer cell-derived EVs would occur through the activation of TLR2/NF-κB signaling by the interaction of EVs surface heat shock protein 70 (HSP70) with cells (Li et al., 2016). In a different experiment, EVs from KMBC (human cholangiocarcinoma cells) enhance modifications of MSCs toward a fibroblastic phenotype. Such transformation is not the only change: MSCs brought into contact with KMBC-EVs subsequently secrete IL-6 which causes an increase in cancer cells proliferation (Haga et al., 2015). Chronic myelogenous leukemia (CML) uses the same pathway to spread. CML-EVs interact with BM-MSCs and this stimulation initiates IL-8 production by MSCs which permits CML cells proliferation (Corrado et al., 2014). The cross-talk between MSCs and cancer cells leads to a specific sequence. First, cancer cells interact with MSCs via their EVs to modulate them and in response, the modified MSCs participate in cancer progression trough their own EVs (Corrado et al., 2014; Haga et al., 2015).

## Conclusion

Cellular communication by EVs relies on the nature of donor cells and recipient cells (Figure 1). Research on MSC-EVs is prolific and shows their potential in regenerative medicine, whereas there is less literature about EVs originated from healthy differentiated cells. For these last, the difficulties may originate from a low proliferation and a poor EV production *in vitro*. In the case of cancers, the mechanisms of the cross-talk between the tumor and MSCs are yet not fully unraveled, but some works on EVs as therapeutic cargo show promising results.

## Author contributions

GD discussed the work and wrote the manuscript. BM helped to write the manuscript and designed the figure. PM helped to discuss the work and to edit the manuscript. EV drafted the manuscript, discussed the work, edited the manuscript, and gave the final approval of the version to be published.

## Funding

GD and BM are supported by a Ph.D. scholarship granted by the French Ministry of National Education, Higher Education and Research (MENESR). EV was supported by grants from the Conseil Régional de Lorraine in association with the Pacte Lorraine (RNG14XNF-AOP14) and the Fédération de Recherche 3209 BMCT (Project RN1PFEAS-AOP15-EXOSEQ).

### Conflict of interest statement

The authors declare that the research was conducted in the absence of any commercial or financial relationships that could be construed as a potential conflict of interest. The reviewer DT and handling Editor declared their shared affiliation, and the handling Editor states that the process nevertheless met the standards of a fair and objective review.
